# Comparison of Two Different Methods for ProSeal^TM^ Laryngeal Mask Fixation

**DOI:** 10.4274/TJAR.2023.231225

**Published:** 2023-10-24

**Authors:** Funda Atar, Gülsen Keskin, Filiz Karaca Akaslan, Yasemin Tıraş, Aslı Dönmez

**Affiliations:** 1 Department of Anaesthesiology and Reanimation, Etlik City Hospital, University of Health Sciences Turkey, Ankara, Turkey

**Keywords:** Airway management, fiberoptic bronchoscopy, fixation, laryngeal mask airway, LMA displacement

## Abstract

**Objective:**

This prospective randomized study compared 2 different methods for Proseal^TM^ Laryngeal Mask Airway (PLMA) fixation.

**Methods:**

Patients scheduled for ureterorenoscopic lithotripsy surgery in the lithotomy position were included in the study. General anaesthesia with PLMA was administered to the patients. To achieve PLMA fixation, patients were randomly assigned to either adjustable elastic band (Group I) or adhesive tape fixation (Group II). Fiberoptic bronchoscope (FOB) evaluation and glottic image grading (grade 1-4) and lip margin distances of PLMA (M1 and M2) were evaluated before and after the surgical procedure.

**Results:**

We enrolled 116 patients. Surgery of 7 patients was postponed. PLMA dislocated in 2 patients in group II during positioning. For another patient who used adhesive tape in Group II, it was removed because it could not adhere to properly, and a new sticking plaster was used. The study was completed with 106 patients. In FOB evaluation, the number of patients with optimal FOB grade (FOB grade 1) after PLMA was inserted and fixed was more in Group I than in Group II (*P* = 0.01). FOB evaluation was repeated at the end of the operation, and the number of patients with the worst FOB grade (FOB grade 4) was 0 (0%) and 11 (10.5%) in Groups I and II, respectively. PLMA displaced more than 1 cm in 10 (18.9%) patients in Group I and in 30 patients (56.6%) in Group II.

**Conclusion:**

The adjustable elastic band method is simple, easy, and convenient and can be used in any surgical procedure for PLMA fixation.

**Main Points**

• Inappropriate fixation of the Proseal^TM^ Laryngeal Mask Airway (PLMA) poses a threat to airway-related complications, such as gastric insufflation, regurgitation, aspiration of gastric contents, and hypoxemia.

• This study presents the fixation method we developed for PLMA.

• Adjustable elastic band reduces PLMA movement and prevents displacement.

• The adjustable elastic band method is simple, convenient and is superior to adhesive tape in patients with traumatized skin, edentulous mouth, or beard.

## Introduction

The laryngeal mask airway (LMA) is a useful and safe device in modern anaesthesia. Although LMA insertion is more manageable than an endotracheal tube in various studies, the success rate is 80.6% for the first attempt.^1^ Inappropriately placed LMAs pose a threat to airway-related complications, such as gastric regurgitation, aspiration, and hypoxemia.^2^ Although the incidence of intraoperative displacement of the first-generation LMAs is 26.7%, no data were found regarding the second-generation LMAs.^3^

The gastric drainage tube and bite block in Proseal^TM^ LMA (PLMA; Intavent Orthofix, Maidenhead, UK), a second-generation LMA, are reported to provide an advantage in keeping it in stable position.^4,5^ Appropriate fixation of the PLMA can reduce the risk of device displacement with changes in patient position, especially the head and neck. Different LMA fixation approaches, such as adhesive tape, bandages, and umbilical tape, are used with it.^6,7^ Inappropriate fixation may cause regurgitation, displacement, and undesirable adverse effects due to adhesive tape or ligation.

Successful PLMA insertion is primarily evaluated clinically, given suitable and sufficient chest excursion, using a capnogram, without a leak at a peak inspiratory pressure of 20 cmH_2_O.^2^ However, these clinical signs do not guarantee correct positioning and continuity. The direct visual technique using a fiberoptic bronchoscope (FOB) is considered a better alternative for placement than the classic method.^2,8,9,10^

Our study compared 2 different PLMA fixation methods with FOB examination.

## Methods

The study was approved by the Clinical Research Ethics Committee of Dışkapı Yıldırım Beyazıt Training and Research Hospital, University of Health Sciences Turkey (ref: 134/13) on April 4, 2022. Written informed consent was obtained from all patients participating in the trial. In addition, this trial was registered at ClinicalTrials.gov (NCT05433740). This prospective randomized study was conducted between 30 August 2022 and 30 September 2022.

In this prospective randomized single-center study, we included American Society of Anesthesiologists (ASA) I-III patients with Mallampati scores I-II, and ≥18 years of age who underwent elective ureterorenoscopic (URS) lithotripsy surgery. We chose URS to observe the risk of PLMA displacement due to the patients being placed in the lithotomy position. The exclusion criteria were: risk of regurgitation or aspiration (e.g., dysphagia), pulmonary diseases (e.g., chronic bronchitis), body mass index (BMI) of ≥35 kg m^2-1^, head and neck anomalies, neck movement limitations, inability to open mouth, obstructive sleep apnea, abnormal or loose teeth, mandibular joint movement limitation, and beard.

The patients were transferred to the operating room without premedication. Standard monitoring included noninvasive arterial blood pressure, electrocardiography, and peripheral O_2_ saturation. Pre-oxygenation was performed with 100% O_2_ with tidal-volume ventilation for 3 min. Induction was performed with intravenous fentanyl 1 µg kg^-1^ and propofol 2 µg kg^-1^. A neuromuscular blocker was not administered. PLMA size was determined on the basis of patient weight. PLMAs were lubricated using a water-soluble gel and inserted using the index finger. A maximum of 3 attempts were allowed. After 3 failed attempts, the airway was secured as per the decision of the anaesthesiologist. These patients were excluded from the study. PLMA cuffs were inflated as recommended by the manufacturer. Patients were randomized into two groups using the closed envelope method. PLMA was fixed with an adjustable elastic band we designed for Group I and adhesive tape for Group II.

**Figure 1:**
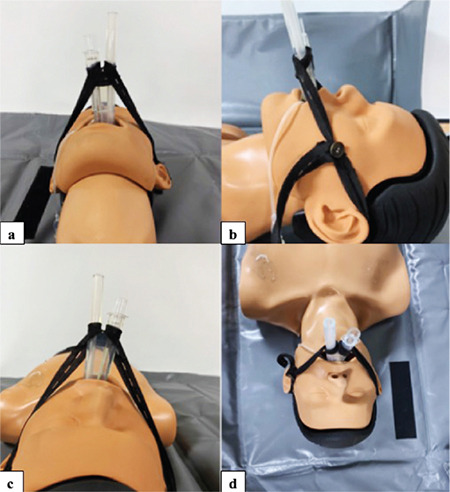
Application of adjustable elastic band method on mannequin; a) Bottom view of adjustable elastic band method, b) Left side view of adjustable elastic band method, c) Top view of adjustable elastic band method, d) Front view of the adjustable elastic band method.

**Figure 2:**
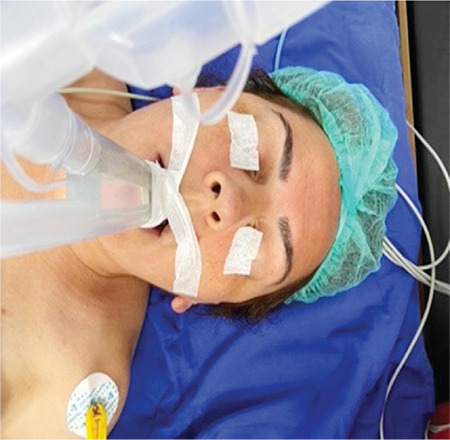
Fixing the PLMA with the adhesive tape method. PLMA, Proseal^TM^ Laryngeal Mask Airway.

Following LMA insertion, placement was confirmed with clinical tests (chest and bag movement with ventilation, no leak at 20 cmH_2_O of airway pressure, and capnogram). Afterwards, LMA was fixed according to the group selection while the patient was in the neutral position. The adjustable elastic band was for single use. With this method, a lacing strap with a button at one end and buttonholes along the band are looped around the bite block section of the outer end of the PLMA. The ends were brought between the tubes over the outer end of the bite block, adjusted at or above the ear (excluding neck vessels), and secured by buttoning in the appropriate hole (Figure 1). In Group II, adhesive tape was fixed to the maxilla (Figure 2). FOB (Karl Storz /Germany, Tuttlingen, Germany, 11302BD2) evaluation and glottic image grading (grade 1-4) and lip margin distances of PLMA (M1 and M2) were assessed before and after the surgical procedure. The same anaesthesiologist performed all PLMA insertions and fixations. However, it was a second anaesthetist who did the FOB review. All fiberoptic evaluations were performed while the tip of the FOB was 1 cm at the end of the ventilation port of the PLMA. The position of the LMA was graded as per the fiber optic scoring system described: 1-glottis seen, 2-epiglottis and glottis seen, 3-epiglottis impinging on the grille, glottis seen, 4-epiglottis downfolded, glottis not seen.^11^ After proper placement and fixation of the LMA, the FOB grade was recorded (G1). Then, the lip level measurement of PLMA (M1) was recorded. To avoid the weight of the anaesthesia circuit, the y part of the circuit was connected to the shield separating the anesthesia and surgical areas. Patients were placed in the lithotomy position for surgery. Patients were ventilated with a tidal volume of 6-8 mL kg^-1^ at a rate of 10-14 breaths min^-1^ to maintain ETCO_2_ between 35 and 40 mmHg. Anaesthesia was maintained with 2 to 2.5% sevoflurane in an oxygen-air (50-50%) mixture. For perioperative analgesia, 0.05 to 0.1 µg kg^-1^ min^-1^ remifentanil infusion was used. PLMA was removed and reinserted in case of a leak at 20 cmH_2_O of airway pressure and absence of capnogram, if there was no chest and bag movement with ventilation during the surgery. These patients were excluded from the study. At the end of the surgery, before awakening the patient, on the same anaesthetic depth, after the operating table was taken to the neutral position, FOB grade (G2) and PLMA lip level (M2) measurements were repeated. FOB grade changes between 2 measurements were calculated as G2-G1. The displacement of the PLMA was measured by the difference between M2 and M1.

Age, gender, BMI, ASA score, comorbidity, number of LMA insertion attempts, hemodynamic data, leak volume, peak airway pressure values, FOB grades, PLMA lip alignment levels, and complications related to fixation were recorded for all patients. The difference between the set tidal and exhaled volumes gave the leak volume. Surgery completed, lithotomy position turned to normal position, measurement of the anesthesia team (G2 and M2) done, and after that anesthesia discontinued.

No previous study has used different PLMA stabilization techniques, so the necessary sample size for research was determined with the G*Power 3.1 (Faul, Erdfelder, Lang, & Buchner, 2007) program before data collection. The minimum sample size was estimated to be 53 patients for each group, with an effect size of 0.5, a power of 80%, and a type I error of 0.05.^12^

### Statistical Analysis

Statistical analysis was performed using IBM SPSS Statistics for Windows, v. 25.0 (IBM Corp., Armonk, N.Y., USA). Normality assumptions of the data were checked by the Kolmogorov-Smirnov test. Descriptive statistics were presented mean ± SD, median (IQR), frequency (n), and percentage (%) for numerical variables. For the data analysis, an independent 2-group t-test (Student’s t-test) was used to compare the two groups, and the Mann-Whitney U test was used when prerequisites were not met. Categorical data were analyzed using Fisher’s exact and chi-square tests. After PLMA insertion and removal, parameters were compared for paired ratios using the McNemar and Wilcoxon signed rank tests. *P* < 0.05 was considered to be the statistically significant for these tests.

## Results

**Figure 3:**
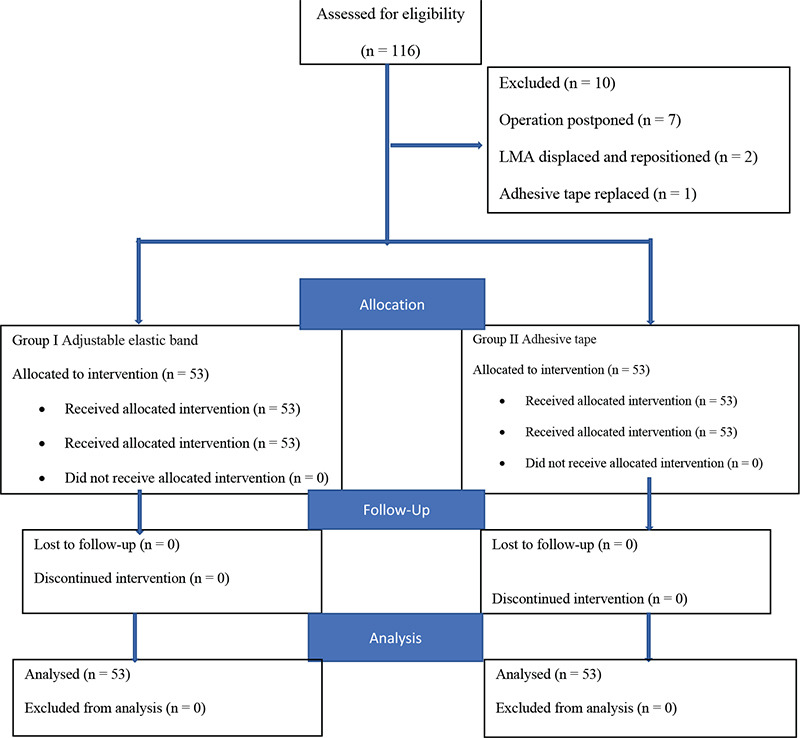
Flow diagram of patients recruitmens.

**Figure 4:**
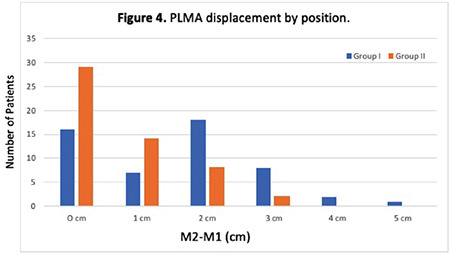
LMA displacement by position. LMA, laryngeal mask airway; PLMA, Proseal^TM^ Laryngeal Mask Airway.

A total of 116 patients, 58 per group, were registered to allow for dropouts (Figure 3). The surgery of 5 patients in Group I and 2 in Group II was postponed. In two patients in Group II, LMA was displaced and repositioned in attempting to position the patient. For another patient who used adhesive tape in Group II, it was removed because it could not adhere to properly, and a new sticking plaster was used. These patients were excluded; therefore, the study was completed with 106 patients. No intergroup differences were observed in terms of demographic data, ASA classification, and comorbidities. The duration of operations was 48±16.1 min and 47.5±23.5 min for Group I and Group II, respectively (*P*=0.753) (Table 1). There were no differences in hemodynamic parameters, peak airway pressures, and leak volumes among groups (*P* > 0.05, Table 1). In the FOB evaluation, the vocal cords were more visible in Group I than in Group II at the time of insertion (*P*=0.01) (Tables 2 and 3). At the end of surgery the FOB evaluation was repeated, and we found that the epiglottis was downfolded in 0 (0%) and 11 (10.5%) patients in Groups I and II, respectively (Tables 2 and 3). The G2-G1 difference was significantly higher in Group II than in Group I (86.8%, 41.5%, *P* < 0.001, respectively) (Table 4). The PLMA displacement distances for each fixation method are graphically shown in Figure 4. The adjustable elastic band significantly reduced PLMA movement compared with adhesive tape. Thirty patients (46.6%) experienced more than 1 cm PLMA movement when adhesive tape was used to secure the LMA against 10 (18.9%) when an adjustable elastic band was used (*P* < 0.001). No complications were observed in patients who used adjustable elastic bands.

## Discussion

In this study, we compared the adjustable elastic band that we designed for PLMA fixation with adhesive tape. This band significantly reduced the mobility of the PLMA and provided better fixation than the adhesive tape.

Studies have emphasized that proper initial LMA insertion and fixation are important in maintaining the LMA position.^8^ In our study, even at the fixation stage, the visibility of the vocal cords was better after PLMA insertion with adjustable elastic band fixation than that with adhesive tape. For proper placement, the distal end of the LMA must fit tightly against the upper esophageal sphincter (UOS).^7^ Inward force with PLMA fixation reduces the possibility of extrusion and misplacement.^7^ As such, the correct approach for fixing the PLMA in place is to apply the ends of the adhesive tape to the maxilla.^7^ Our study fixed the PLMA to the maxilla in the adhesive tape group. However, when evaluated with FOB, we observed that the vocal cords were visible in only 56.6% of patients with adhesive tape, whereas visibility was 81.1% with the adjustable elastic band method. These results suggest that the adjustable elastic band method is more effective for PLMA fixation. Studies investigating ideal positioning of LMA by FOB in children show that although ventilation is clinically normal, only 12 to 50% of LMAs are properly positioned.^13,14,15^ These studies did not investigate different fixation methods but only different LMA placement methods. They used adhesive tape for all patients. As a result, we think the bands may not have exerted pressure in the required inward direction to ensure that the distal end of the mask was pressed against the UOS.

In our study, we observed that the fiberoptic view changed less in the adjustable elastic band group than in the adhesive tape group at the end of surgery. All patients’ vocal cords were visible in the adjustable elastic band group after surgery. However, visibility of the vocal cords could not be attained with FOB in 11 (10.5%) patients in the adhesive tape group, suggesting that the elastic band does not fully prevent displacement: deviation of the LMA cuff to one side can cause this issue.^3^ However, there were no symptoms of leakage, and ventilation was optimal in both groups. Chandan et al.^10^ reported that ventilation was clinically optimal in all patients, although the cuff position was optimal in only 56.7% of patients at the time of insertion. Another study examined how head and neck position affected the cuff position and oropharyngeal sealing pressure of the LMA in children; it was observed that airway patency was not adversely affected in 97% of patients.^16^ However, complete or partial obstruction of the glottic aperture by the epiglottis might result in increased work of breathing, especially in spontaneous breathing cases or children.^17^ Although the FOB view changed within groups, no audible leak was detected at a pressure of 20 cmH_2_O, and no negative effect on ventilation was observed. Nevertheless, these patients were adults, and spontaneous breathing was not permitted. We cannot generalize the results of this study to spontaneously breathing adults or children, since hypopharyngeal muscular tension can alter LMA positioning; it may be thought that adequate clinical ventilation parameters do not indicate an anatomically or properly placed LMA.

In our study, the adjustable elastic band reduced extreme (>1 cm) PLMA movements, in contrast to the adhesive tape. During anaesthesia maintenance, PLMA ordinarily provides an appropriate airway; position adjustment is infrequently necessary. Nevertheless, displacement may occur, especially if anaesthesia becomes light, the patient moves, or the surgical position changes. Major intraoperative LMA displacement is not frequent, but minor events can occur; which can cause regurgitation, aspiration, or partial laryngeal obstruction.^15^ When positive pressure ventilation is used, the increased airflow resistance may lead to higher airway pressure and opening of the UOS, increasing the risk of regurgitation.^13^ Thus, it can be assumed that the fixation method affects the major or minor displacement of the PLMA.

Inappropriate fixation of the PLMA can lead to complications such as device displacement, increased work of breathing, hypoxemia, gastric inflation, regurgitation, and aspiration. In this study, no complications related to the fixation method were observed in the adjustable elastic ligament group. In the adhesive tape group, for 3 patients, the PLMA was removed from the patient during positioning or the sticking plaster was repeated.

This may be caused by contamination of the adhesive tape by patient secretions or by the weight of the breathing circuit and loosening of the tape. Studies comparing endotracheal tube fixation methods have shown that adhesive tape may not provide adequate protection for unintentional extubation.^18,19,20,21,22^ There are case reports about LMA fixation methods in the literature, but we could not find studies comparing them. Our study found that the adjustable elastic band did not allow outward displacement of the PLMA and fixed it more securely than the adhesive tape. Adhesive tape allergy, burned, traumatized or loose skin, edentation of the mouth as in the elderly, or facial hair may limit the use of adhesive tape for PLMA fixation.^6^ Forces applied to a taped PLMA deform and pull facial tissues, causing important PLMA movements without adhesive failure.^22^ This may cause displacement of the PLMA. PLMA secured with adjustable elastic ligament was fixed between bony structures that did not move under such loads, while protecting venous neck structures. We believe that this may be an advantage in patients with loose skin, edentulous mouth, or beards. Along with avoiding the disadvantages of adhesive tape, we found that this method offered better control over the applied pressure.

### Study Limitations

The inability to hide the PLMA fixation method from the observer can be considered a limitation of our study. In addition, this method may not be applicable to every patient due to economic reasons and the lack of materials. Observing only patients in the lithotomy position is another limitation. The positive contribution of this fixation method to PLMA mobility can be supported by creating different surgical positions, longer surgery times, or patients with different BMIs.

## Conclusion

To our knowledge, this is the first study to compare LMA fixation methods. Our results indicate that the adjustable elastic band reduces PLMA movement and may prevent displacement. The adjustable elastic band method is simple, easy, and convenient and can be used in any surgical procedure for PLMA fixation. In addition, we believe that the method is superior to adhesive tape in patients with adhesive tape allergy, burnt or traumatized skin, edentulous mouth, or beard, and in cases in which PLMA fixation can be challenging due to blood, sweat, mouth, and facial secretions. In addition, this method can make a significant contribution in cases where the lithotomy position or the table position is frequently changed during the procedure.

## Figures and Tables

**Table 1:**
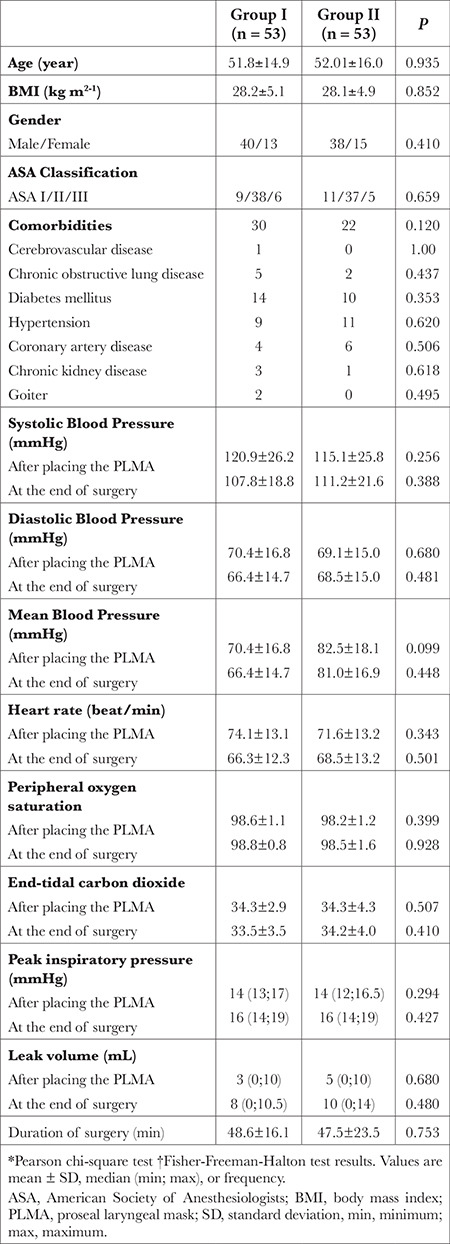
Characteristics of Patients, Comparison of Hemodynamic Parameters, and Characteristics of Successful Airway Insertion (n = 106)

**Table 2:**
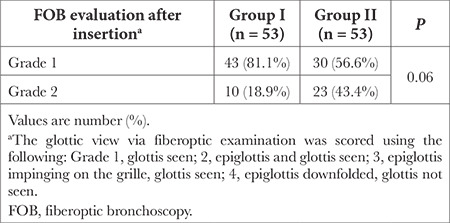
FOB Evaluation After Insertion Within the Groups

**Table 3:**
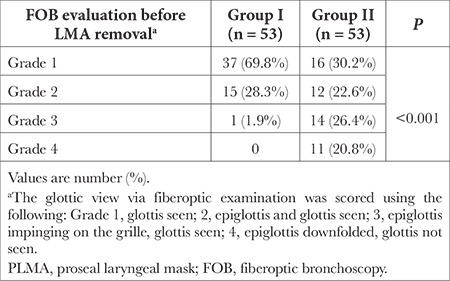
FOB Evaluation Before LMA Removal Within the Groups

**Table 4:**
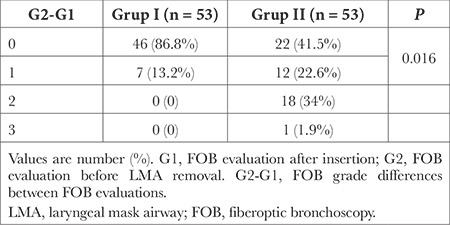
Comparison of FOB Grade Within the Groups
